# Necrophilic behaviour in wild stump-tailed macaques (*Macaca arctoides*)

**DOI:** 10.1038/s41598-024-61678-z

**Published:** 2024-05-13

**Authors:** Aru Toyoda, André Gonçalves, Tamaki Maruhashi, Suchinda Malaivijitnond, Ikki Matsuda

**Affiliations:** 1 Japan Society for the Promotion of Science, Chiyoda-ku, Tokyo 102-0083 Japan; 2 The Japan Monkey Centre, Inuyama, Aichi 484-0081 Japan; 3 National Primate Research Center of Thailand, Chulalongkorn University, Saraburi, 18110 Thailand; 4 Wildlife Research Center of Kyoto University, 2-24 Tanaka-Sekiden-cho, Sakyo, Kyoto 606-8203 Japan; 5 Section of Cognitive Neuroscience, Center for the Evolutionary Origins of Human Behavior, Kyoto University, Sakyo, Japan; 6 Musashi University, Nerima, Tokyo 1768534 Japan; 7 Department of Biology, Faculty of Science, Chulalongkorn University, Bangkok, 10330 Thailand; 8 Chubu Institute for Advanced Studies, Chubu University, Kasugai, Aichi 4878501 Japan; 9 Chubu University Academy of Emerging Sciences, Kasugai, Aichi 4878501 Japan; 10 Institute for Tropical Biology and Conservation, Universiti Malaysia Sabah, Jalan UMS, 88400 Kota Kinabalu, Sabah Malaysia

**Keywords:** Thanatology, Reaction to death, Necrophilic behavior, Macaque, Animal behaviour, Anthropology, Sexual selection, Behavioural ecology

## Abstract

Necrophilic behavior (attempted copulation with corpses) has been scarcely reported in non-human primates, especially in the wild. Here is the first case of necrophilic behavior observed in wild stump-tailed macaques in Thailand. Six groups of total N > 460 individuals have been identified and habituated. The corpse of an adult female was found and directly observed for 2 days and by camera trap for 3 days. The cause of death could not be identified, but no prominent physical injury was detected. Within 3 days of the observation, three different males attempted copulation with the corpse. Noteworthy for this observation was that not only males in the group of the dead female but also males from different groups interacted with the corpse. Taken together, these observations suggest that some cues emanating from the corpse coupled with a nonresistant/passive orientation may have triggered these responses in the males. Given that necrophiliac responses have been scarcely reported in non-human primates, our findings provide new insight into these behaviors and to comparative thanatology in general.

## Introduction

Death is an inevitable phenomenon universally experienced among living organisms^1^. The newly emerged field of comparative thanatology is at its center, tasked with elucidating how non-human animals perceive and respond to death and death-related stimuli^2,3^. Research within this discipline is both involved in reporting anecdotes and tasked with systematically analyzing the various reactions that animals (including non-human primates, cetaceans, and elephants) exhibit when confronted with dead conspecifics and heterospecifics. Interactions with dead conspecifics are usually defined as expressions of interest or interaction with the deceased; these have been termed morbidity/thanatological interactions^4,5^. They may encompass physical interactions (touching, grooming, hitting, dragging, etc.) and vigils (prolonged proximity) and visitations; some striking examples in the literature include the carrying of dead offspring^6–8^, aversion towards death-odors^9^, and interest in conspecific skulls^10^.

Various explanations have been proposed for the behavior of nonhuman primates, particularly in relation to the carrying of dead infants. For example, it may be because these animals with dead infants do not understand the concept of death^11^, or they use the dead infants as a tool to gain an advantage in their relationships with other individuals^12^, or they reduce their grief over death by carrying dead infants^13^, or they spend time with a dead infants to gather information about death with learning about it^14^.

Curiously, however, reports on necrocoitus in non-human primate remain extremely scarce although several vertebrate taxa engage in it; including amphibians, reptiles, birds and mammals^15–17^. Therefore, the primary focus of this paper is on the phenomenon of necrophilia (also known as Davian behavior) in wild primates. While sexual behavior towards deceased conspecifics in non-human animals has long fascinated researchers in ethology and behavioral ecology, it has seldom been linked to comparative thanatology, despite being a facet that warrants deeper exploration and integration.

Delving into the extensive published occurrences in non-human animals on this topic, i.e. necrocoitus, we can discern three main hypotheses from the literature:

1. Breeding season hypothesis: the increased incidence of necrophilic responses aligns with the breeding season, suggesting a correlation between it and higher male hormone levels during this period^18,19^.
2. Passive female hypothesis: the passive posture of a dead female, triggers necrocoital actions, influenced by visual cues such as posture, and form, suggesting that visual and behavioral cues play a significant role in motivating males^20,21^.
3. Peripheral male hypothesis: Instances involving young reproductive males or older non-breeding males struggling to access partners due to hierarchical or social positions reported in the literature, may be a manifestation of challenges faced by peripheral males in securing mating opportunities leading to sexual interactions with deceased individuals^22^.

Other alternative explanatory hypotheses have involved emotional distress (observed necrophilic behavior in animals stems from emotional distress, possibly linked to social contexts and necrocoitus could serve as an unconventional outlet for emotional turmoil) and dominance display (necrophilic behavior functions as a display of dominance, indicating individuals use it as a strategy for asserting control or reinforcing their social hierarchy and establish dominance within the group).

In this context, we report the details of sexual behaviors towards a dead conspecific in wild stump-tailed macaques (*Macaca arctoides*) and discuss the implications of such behavior, in light of the above-stated hypotheses.

## Methods

Since 2015 we have intermittently conducted more than 1000 days of observation and recorded the behaviors of stump-tailed macaques in the Khao Krapuk Khao Taomor non-hunting area, Phetchaburi Province, Thailand (12° 47′ 59″ N, 99° 44′ 31″ E). The study site is home to six multi-male/multi-female social groups of stump-tailed macaques (group sizes range from 41 to 120 individuals) totaling more than 460 individuals. The study population has been named as Ting-Group, Nadam-Group, Third-Group, Fourth-Group, Wngklm-Group, and Ruay-Group, and > 80% of individuals have been identified^23^.

In the case of this observation, all behaviors were recorded using a video camera (SONY FDR-AX60) for a series of actions from immediately after the discovery of the corpse until contact was initiated by another individual. In addition, a camera trap (HykeCam SP2) was used from January 31st to February 1st, 2023 for monitoring while the observer was not present at the site. The camera trap was equipped with a motion sensor and was set to automatically begin recording when an animal appeared in the vicinity of the corpse.

On January 30th, 2023, at around 18:00, while observing the Third-Group, a corpse of the adult female of Ting-Group, identified as TNG-F11, was found on the ground near a temple within the home range of animals. This place is where Buddhists visiting the temple often feed the monkeys and is also a resting site for them. The observer, AT, confirmed that the female was definitely dead, and henceforth this female was referred to as a corpse. Given the absence of visible physical injuries, it could be inferred that a predator attack or fighting amongst conspecifics were unlikely causes for death. Since this female was recorded as being weak (her face was pale and her movements were sluggish) on January 29th, we assume that the cause of death could be illness.

On January 31st, AT went back to the temple to set a camera trap and found that a temple monk was going to bury a corpse as common practice. AT explained the purpose of the observation to the monk, and he allowed to leave the corpse there for an additional day. Therefore, a camera trap was placed in front of the corpse to record the detailed responses toward the corpse from other individuals until February 1st.

### Ethics approval

All data acquisitions and procedures during the fieldwork were approved by the National Research Council of Thailand (#0002/6910) and the Department of National Parks, Wildlife and Plant Conservation of Thailand.

## Results

**Figure 1:**
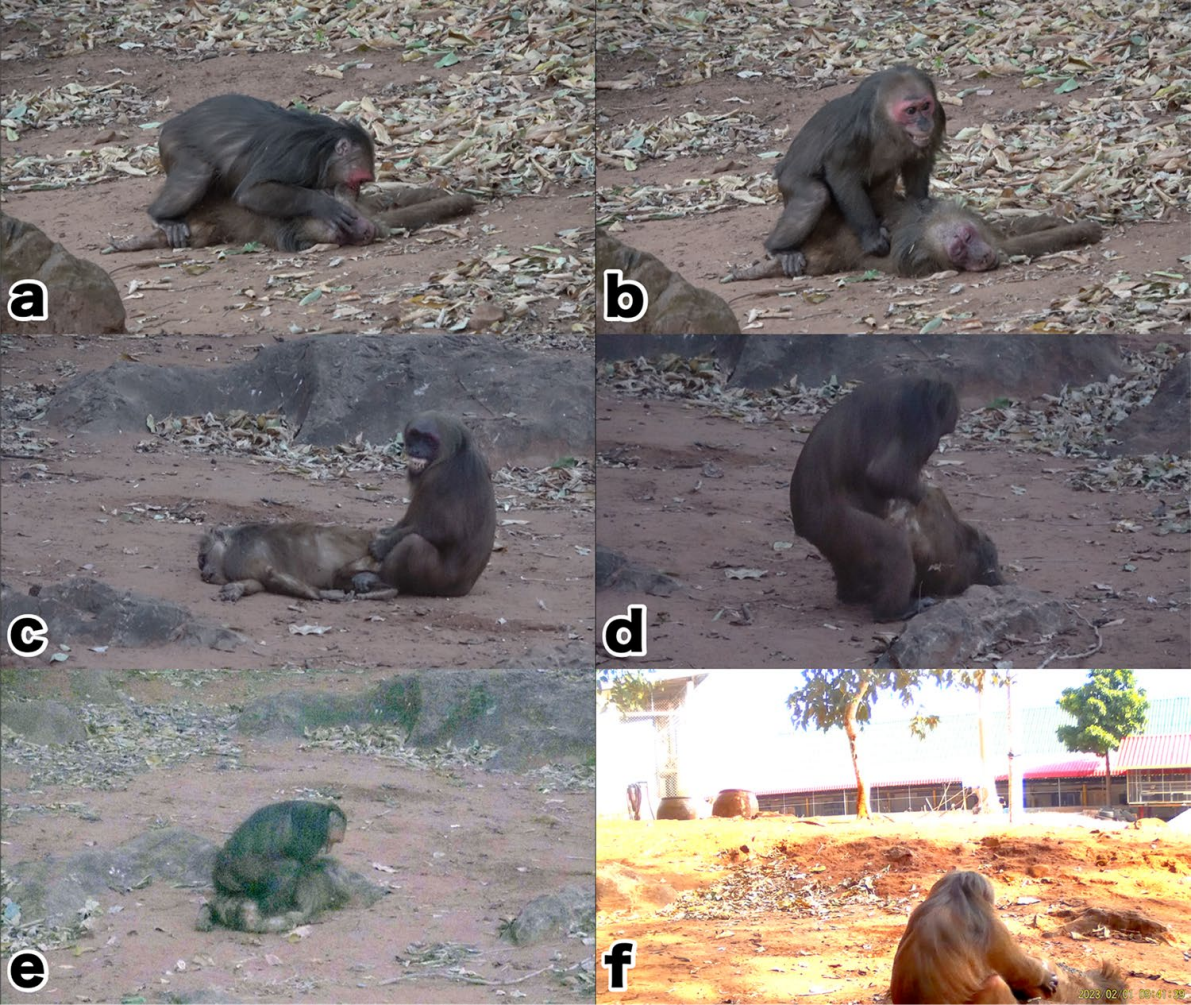
Highlights of four cases of necrophilic behavior observed. (**a**) and (**b**) are Case#1, (**c**) and (**d**) are Case#2, (**e**) is Case#3 and (**f**) is Case#4: (**a**) TRD-M48 looking into the face of the corpse (TNG-F11) and thrusting with a bite. (**b**) TRD-M48 showing Teeth-chattering, a facial expression commonly seen during copulation, during thrusting. (**c**) TNG-M13 initiating copulation while Teeth-chattering after vaginal inspection behavior towards the corpse. (**d**) TNG-M13 lifting its hips to hold the corpse in position for copulation (this behavior was also recorded in Case#4). (**e**) NDM-M49 thrusting across the corpse's back, confirming that no vaginal penetration had taken place. (**f**) Video footage taken by camera trap. Numerous flies can be seen swarming the corpse. This male was identified as TNG-M13 based on the physical characteristics.

*Case #1 at 18:15, on 30th January by direct observation*: an adult male of Third-Group approached the corpse, and shortly after vaginal inspection, the male groomed the corpse for 30 s and then left. One juvenile male was watching the activities one meter away. After the juvenile male left the corpse, an adult male (TRD-M48; peripheral male) and two juvenile males of Third-Group approached the corpse. Two juvenile males sniffed, groomed and looked into the face of the dead female, though TRD-M48 briefly groomed around the buttocks and abdomen, then TRD-M48 pulled the corpse to him and then initiated copulation after vaginal inspection. During thrusting, the male looked into the dead female’s face and bit her face (Fig. 1a,b). After 28 s of thrusting, the male ejaculated (this male's ejaculation was confirmed by pulling a solidified semen out of his penis and eating it after he left the corpse) and kept “Pair-sit” posture for about 60 s, then left the female. All other members of the Third-Group had already started foraging and the male was in last place. After this copulation, no individuals of the Third-Group approached to the corpse.

*Case#2 at 18:14, 31st January by direct observation*: the Ting-Group came to the temple and encountered the corpse. As the dead female had belonged to this group, more individuals gathered around the corpse as compared to the responses of the Third-Group members on January 30th. During the first 13 min of recording, 30 members including 10 males, actively contacted to the corpse mainly through grooming. After 13 min in the recording, an adult male (TNG-M13; peripheral male) approached the corpse. The male looked into the face and sniffed the corpse, and then performed vaginal inspections several times. After chasing away other individuals around the corpse, the TNG-M13 turned the lying corpse on its back and copulated (Fig. 1c). The first copulation resulted in ejaculation after 36 s of thrusting and kept “Pair-sit” for 42 s. Ten seconds later, the same male TNG-M13 copulated again, but he only maintained the posture with the penis inserted for 30 s and then disengaged from the female without ejaculation. Ten seconds later, TNG-M13 again initiated vaginal inspection behavior, lifting the corpse’s buttocks (Fig. 1d) and turning her around, then started the copulation. However, TNG-M13 maintained the posture with the penis inserted for 10 s then he stopped copulating without ejaculation. After 53 s, the male copulated again by lifting the buttocks of the corpse for 15 s, but he stopped interaction. None of these three copulations resulted in ejaculation, though after those three attempts, the male finally started copulating a fourth time and ejaculated after 25 s of thrusting. After ejaculation, the male maintained “Pair-sit” for 2 min and then left the place.

*Case#3 at 18:34, 31st January by direct observation*: After Ting-Group left from the temple, the Nadam-Group came to the temple and encountered the corpse. At least 12 individuals including four males, contacted the corpse through grooming, while many others were only watching the corpse from a distance > 2 m or left the place shortly after sniffing the corpse. After 2.6 min in the first recording, a young adult male named NDM-M49 approached the corpse, sniffed the vagina and then began pulling and grooming around her neck and arm. After 37 s when another adult male sitting next to him, left the place, NDM-M49 male began to copulate; after 15 s of thrusting, the male appeared to ejaculate, but at the same time another unidentified adult male re-approached to NDM-M49 and NDM-M49 moved away from the corpse. After this unidentified male left, NDM-M49 began to copulate again and ejaculated after 21 s of thrusting. During the thrust, NDM-M49 lifted the head of the corpse and looked into her face. After 1.6 min of “Pair-sit”, NDM-M49 left. For this second copulation, the video data suggests that no vaginal insertion was made (Fig. 1e) and it is likely that he was thrusting against the buttocks; two minutes later, this NDM-M49 approached the corpse again, but only pulled the head and left immediately.

*Case#4 at 9:41, 1st February by camera trap data*: AT could not conduct observations this morning, but a camera trap was working to collect the data instead. The camera trap recorded an adult male in contact with the corpse. This group was identified as Ting-Group based on the individuals surrounding, and the male was identified as TNG-M13 based on the physical characteristics. This male was the same male as *Case#2*. After the vaginal inspection, the male appeared to start copulating (Fig. 1f), but it could not be confirmed whether he copulated and ejaculated because he moved out of the camera’s field of view. On this day (the third day post-mortem), the corpse was considerably decomposed and swarmed by a considerable number of flies.

No group came to the temple after this time, and in the evening AT took responsibility for burying this dead female (“burying dead animals” is a common practice by the monks at this temple and we complied with their wishes).

## Discussion

To the best of our knowledge, there are only three published reports suggestive of necrophilia in primates; two reports in captive primates, i.e. hamadryas baboons^24^ and stump-tailed macaques^25^, and one in wild common marmosets^26^. In the marmoset case, this was a record of behavior toward a dying individual, not a corpse. This observation was the first documented necrocoitus in-depth in a wild primate encompassing several important points. First, the three males copulating the corpse were all subordinate and periphery males which had limited access to females^23,27^. There remains a possibility that a male, who usually had limited or no access to females, attempted to mate with her when he encountered a female in a passive or unresisting condition if such an opportunity arose. Second, during her life, this female (TNG-F11) was the most frequently copulating female in the Ting-Group, and therefore was likely the most preferred female for males to mate with, according to the previous observations^23^. This species is generally a non-seasonal breeder^28^, but in the population at this study site, mating frequency is higher during the dry season, around October to February (Toyoda, unpublished data). The observation period of this study between 30th January and 1st February, 2023 fell into the dry season, which would possibly stimulate the subordinate males to copulate with the corpse. Third, although we remain agnostic as to whether these macaques possess a concept of death, the data from our observations seems to show otherwise. These individuals would attempt to copulate even though the corpse was decomposing, emitting a foul odor and swarming with flies. Meanwhile, it is unclear why the higher-ranking males, who would normally occupy the mating opportunity, did not do so. Fourth, the males that copulated with the corpse included males from different groups. This adds another level of complexity to the question about intra- and inter-group variability, in mortuary activity among the living primates^29,30^.

There were no differences in the behavioral elements found in copulatory behavior with the corpse from that with live individuals. As shown in Table 1, the copulatory behavior in this species usually begins with perineal inspection, mounting, thrusting, ejaculation (this species is single-mount-ejaculator; thrusting usually continues through a single uninterrupted mount until ejaculation), and pair-sitting (male holding the female with the penis inserted in the vagina after ejaculation^28^) (Table 1). All of these behaviors were observed in the present observations, and no strikingly different behaviors observed. In this species, female estrous signs are scarcely observed, and active solicitation from females during copulation initiation is also rare. Additionally, approximately 38% of copulations are coercive, initiated by the male towards the female^23^. This kind of male-driven copulating pattern could be the reason why the copulation with a corpse could be completed without lacking the typical sequence of behavioral elements seen in normal copulatory behavior in these observed cases. Males sometimes vocalize during ejaculation^31^, and this frequency of vocalization is related to social ranking^32^; however, in the case of this observation, the males did not produce any vocalization during ejaculation. This suggests that all of the males that mated with the corpse were low ranking/peripheral males^32^. Behaviors such as looking into the female's face and violent reactions to the female (biting, hair pulling, etc.) during the “Pair-sit” phase are also often observed in the normal copulation (Toyoda, unpublished data). Anyhow, despite a lack in relation to the social system underlying the evolution of the coercive, male-driven mating style observed during copulation, such style may be relevant as a factor in triggering the species' specific necrophilic behaviour.

**Table 1:** Ethogram of general copulatory behavior in Stump-tailed macaques.

Behavior	Definition
Genital inspection	Genital or perineal inspection, as also referred to Fooden^28^. The female spontaneously presents her hindquarters to the male, or the male forcibly conducts inspections on the female. The male inserts a finger into the female's vagina or sniffs. In cases of finger insertion, the male smells the odor of the withdrawn finger or licks the vaginal secretions adhering to the fingers
Mount	The male mounts the female from behind in typical cercopithecine dorsoventral fashion, usually gripping the female’s shanks with his feet and her loins with his hands. Mounting is followed by pelvic thrusting and intromission^28^
Ejaculation	Pelvic thrusting usually continues through a single uninterrupted mount until ejaculation is achieved (single-mount ejaculation)^28^
Pair-Sit	Termed as the "post-ejaculatory pair sit"^38^, this behavior involves the continuation of intromission following ejaculation, during which the male assumes a seated position and draws the female onto his lap. Notably, among macaques, the pair-sit behavior appears to be distinctive to this particular species^28^
Teeth-chattering	A variant of the bared-teeth display, characterized by the full opening of the lips and the occurrence of teeth chattering. This behavior is prominently exhibited during consortship, particularly when the male assumes a mounted position on the female^39^
Copulation call	Post-ejaculation, the context-dependent vocalizations emitted by the male. These vocalizations may be segmented into short syllable (goggoggoggoggo) or persist for an extended duration (goooogoooogoooo). Males with higher rankings vocalize at a higher frequency, while lower-ranking males do not emit vocalizations at the time of ejaculation^32^
Sexual harassment	The collective term referring to any behavior directed towards the copulant(s) that commences immediately post-ejaculation, except with the exception of interruptions^40^. Specifically, these behaviors encompass activities such as circling around the pair, engaging in teeth chattering directed at the pair, making physical contact with the pair, pulling the hair of the copulant(s), and slapping the copulant(s)

Although infrequently observed, necrophilic behavior has been documented in several taxa with the majority including several species of amphibians and reptiles^15^, followed by birds^33^, being exceedingly rare in mammals with copulations reported in badgers, squirrels, sea otters and sea lions^22^. While other sexual interactions have been reported with other species of mammals without explicit copulations they have been interpreted as expressions of physiological stress^26^ or hierarchical dominance^34^. A few relevant bird and mammal cases can help reframe our observations. In the case of birds, it has been suggested that necrocoital actions may be induced by visual cues such as the passive posture of the dead female. In fact, experiments employing taxidermy/model specimens have confirmed that the female posture, form, and color can influence the sexual motivation of the males^20,35,36^. On female passivity as a contributing factor; other authors have suggested that males perceive the lack of rejection by the dead females as a catalyst^21^. Additionally, others refer to their seasonality: higher hormone levels among the males could act as a factor for the high incidence of necrophilic responses during the breeding season^16,18,19^. In mammals, Dickerman^37^ mentions the specific posture of a dead squirrel acting as a releasing cue for the live conspecific. Finally, Colombo and Mori^22^ cited instances involving young reproductive males who may struggle to access partners due to their hierarchical or social position and report this behavior in a young sexually European badger towards a road-killed female.

In conclusion, a parsimonious explanation is that males in our study simply sensed the passivity of the female, possibly coupled with some sexually ventilated cue from the female, and copulated with it. Our report indicates that even with the lack of proper contextual and behavioral cues (live typical behavioral input from a conspecific female), the male macaques still exhibited a strong sexual motivation to mate with the female. On the other hand, it cannot be entirely ruled out that the site where the female died was in an open area of the temple and food was occasionally provided the monkeys, causing them to visit the area frequently, and thus potentially resulting in more frequent contact with the corpse by individuals both within and outside the groups. Nonetheless, given that how primates perceive the phenomenon of death is yet to be fully understood^5,29^, our case report is of relevance to comparative thanatology should remain essential to our understanding of the phenomenon of death in animals.

### Supplementary Information

Supplementary Video 1.

## Data Availability

All data regarding this case report is included in the text. See Supplemental Materials for figure and video. Correspondence and requests for full-length video should be addressed to AT.
